# 505. Impact of Remdesivir on SARS-CoV-2 Clearance in a Real-Life Setting: A Matched-Cohort Study

**DOI:** 10.1093/ofid/ofab466.704

**Published:** 2021-12-04

**Authors:** Vincenzo Spagnuolo, Marta Voarino, Marco Tonelli, Laura Galli, Andrea Poli, Elena Bruzzesi, Sara Racca, Nicola Clementi, Chiara Oltolini, Moreno Tresoldi, Patrizia Rovere Querini, Lorenzo Dagna, Alberto Zangrillo, Fabio Ciceri, Massimo Clementi, Antonella Castagna

**Affiliations:** 1 Vita-Salute San Raffaele University; Unit of Infectious Diseases, IRCCS, San Raffaele Scientific Institute, Milan, Lombardia, Italy; 2 Vita-Salute San Raffaele University, Milan, Lombardia, Italy; 3 Vita Salute San Raffaele University; Unit of Microbiology and Virology, IRCCS San Raffaele Scientific Institute, Milan, Lombardia, Italy; 4 Unit of Infectious Diseases, IRCCS San Raffaele Scientific Institute, Milan, Lombardia, Italy; 5 Unit of Microbiology and Virology, IRCCS San Raffaele Scientific Institute, Milan, Lombardia, Italy; 6 General Medicine and Advanced Care Unit, IRCCS San Raffaele Scientific Institute, Milan, Lombardia, Italy; 7 Vita-Salute San Raffaele University; Internal Medicine, Diabetes, and Endocrinology Unit, IRCCS San Raffaele Scientific Institute, Milan, Lombardia, Italy; 8 Vita-Salute San Raffaele University; Unit of Immunology, Rheumatology, Allergy and Rare Diseases, IRCCS San Raffaele Scientific Institute, Milan, Lombardia, Italy; 9 Vita-Salute San Raffaele University; Anesthesia and Intensive Care Department, IRCCS San Raffaele Scientific Institute, Milan, Lombardia, Italy; 10 Vita-Salute San Raffaele University; Hematology and Bone Marrow Transplant Unit, IRCCS San Raffaele Scientific Institute, Milan, Lombardia, Italy; 11 IRCCS San Raffaele Hospital and Vita-Salute San Raffaele University, Milano, Lombardia, Italy

## Abstract

**Background:**

Evidence regarding the impact of remdesivir (RDV) on SARS-CoV-2 viral clearance (VC) is scarce. Aim of this study was to compare VC timing in COVID-19 patients who received RDV with those who did not.

**Methods:**

Matched-cohort study conducted (25 February 2020-15 April 2021) at the IRCSS San Raffaele, Milan, Italy. The study enrolled hospitalized patients with pneumonia and a SARS-CoV-2 positive nasopharyngeal swab (NPS) at admission and at least one NPS during follow-up. Follow-up started at hospital admission and ended at the date of the first negative NPS (within 30 days after discharge). Patients who received RDV (cases) and patients who did not (controls) were matched based on age (±5 years), sex and PaO_2_/FiO_2_ (P/F; ±10 mmHg) values at admission. NPS were analyzed with RT-PCR. Results described as median (IQR) or frequency (%). Time to VC was estimated with Kaplan-Meier curve and compared with log-rank test.

**Results:**

648 patients were enrolled: 216 cases and 432 controls. Patients’ characteristics at admission are reported in Table 1. VC was observed in 490 patients (75.6%) in a median time of 25 (16-34) days. Overall, time to VC was similar in patients receiving or not receiving remdesivir (*p*=0.519). However, time to VC was different when considering both the use of RDV (yes vs no) and age (≤ or > 63 years), as shown in Figure 1A. A significant finding was also observed considering the use of RDV and P/F values at admission (≤ or > 200 mmHg), as reported in Figure 1B. Among the 490 patients who reached VC during follow-up, overall time to VC was similar in patients receiving or not receiving RDV (p=0.075; Figure 2A); however, RDV use was associated with a higher probability of VC in the subgroup of patients with P/F admission values ≤ 200mmHg (p=0.035; Figure 2B), in the age group 55-65 years (p=0.025; Figure 2C) and in patients with comorbidities (p=0.028).

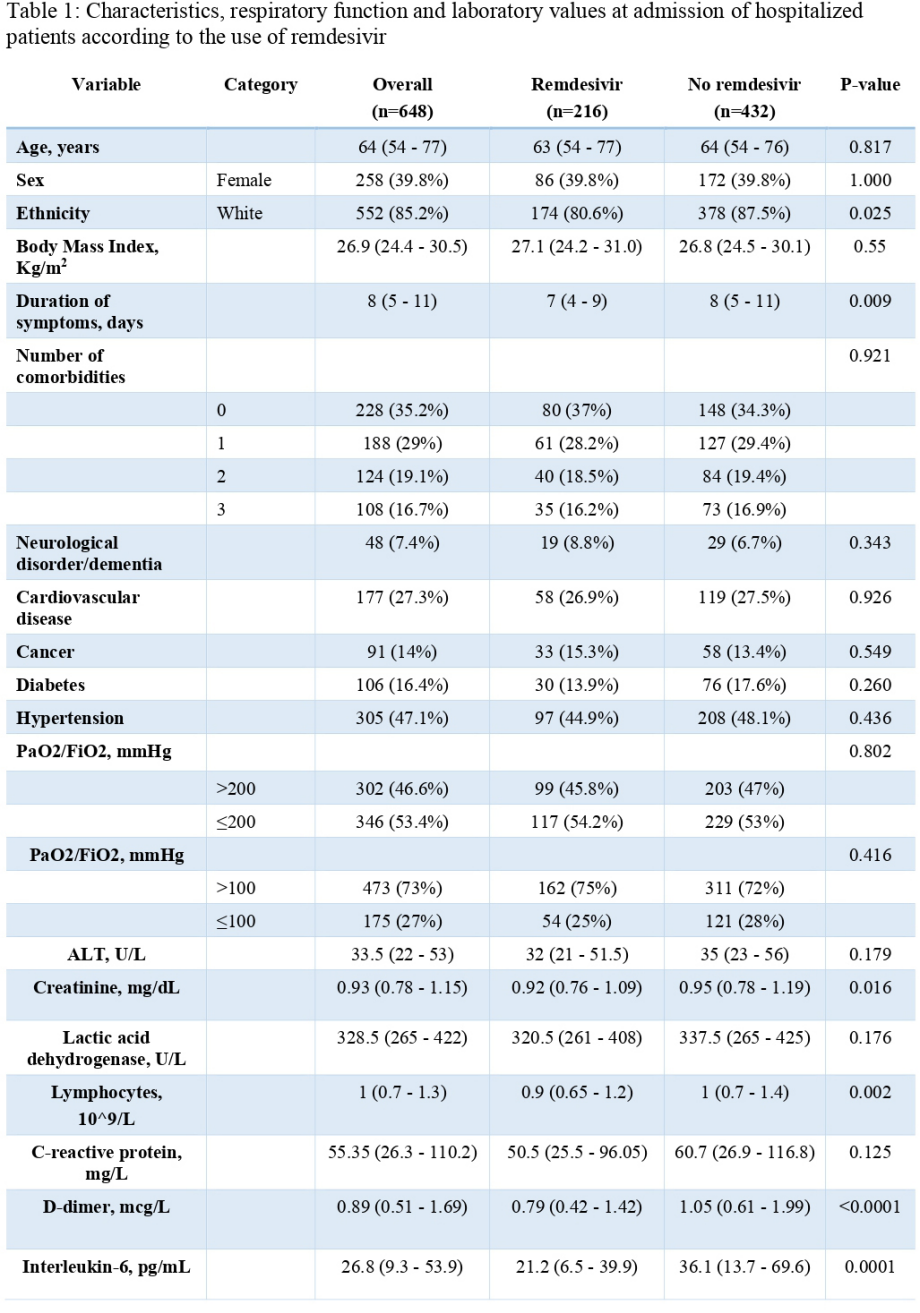

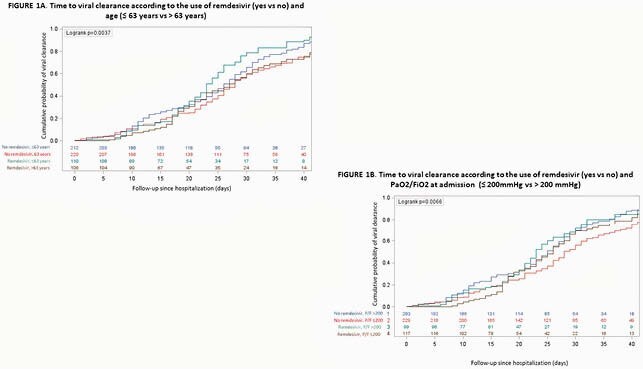

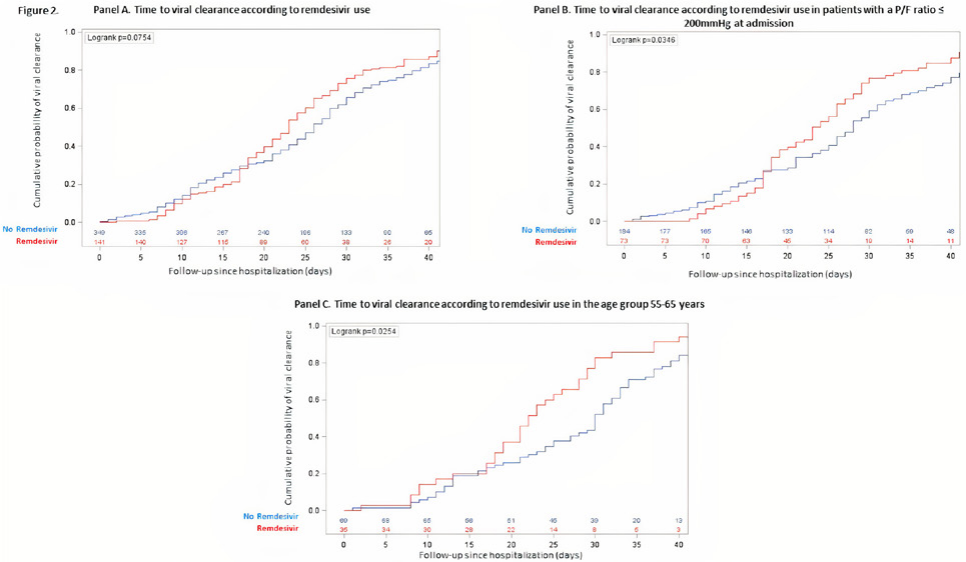

Time to viral clearance among the 490 patients who reached VC during follow-up. Panel A: time to VC according to RDV use. Panel B: time to VC according to RDV and P/F ratio value at admission. Panel C: time to VC according to RDV in the age group 55-65 years.

**Conclusion:**

Time to viral clearance was similar in patients receiving or not receiving remdesivir; however the use of RDV was associated with a benefit on time to viral clearance in younger patients and in those with a P/F ratio at admission ≤200 mmHg.

**Disclosures:**

**Vincenzo Spagnuolo, MD**, **ViiV Healthcare** (Other Financial or Material Support, Preparation of educational material) **Antonella Castagna, MD**, **Gilead Sciences** (Other Financial or Material Support, Speaking fee)**Jansenn-Cilag** (Other Financial or Material Support, Speaking fee)**MSD** (Other Financial or Material Support, Speaking fee)**Theratechnologies** (Other Financial or Material Support, Speaking fee)**ViiV Healthcare** (Other Financial or Material Support, Speaking fee)

